# Prevalence of *Giardia duodenalis* among African children: A systematic review and meta-analysis

**DOI:** 10.1016/j.parepi.2024.e00365

**Published:** 2024-07-04

**Authors:** Sara Kalavani, Sara Matin, Vahid Rahmanian, Ahmad Meshkin, Bahareh Bahadori Mazidi, Ali Taghipour, Amir Abdoli

**Affiliations:** a Zoonoses Research Center, Jahrom University of Medical Sciences, Jahrom, Iran; b Pediatric Department, Jahrom University of Medical Sciences, Jahrom, Iran; c Department of Public Health, Torbat Jam Faculty of Medical Sciences, Torbat Jam, Iran; d Student Committee of Medical Education Development, Education Development Center, Gerash University of Medical Sciences, Gerash, Iran; e Department of Medical Parasitology and Mycology, School of Medicine, Jahrom University of Medical Sciences, Jahrom, Iran

**Keywords:** *Giardia duodenalis*, Children, Africa, Meta-analysis

## Abstract

**Background:**

*Giardia duodenalis* (*G. duodenalis*) is one of the major causes of diarrhea among children. We performed a systematic review and meta-analysis to assess the prevalence of *G. duodenalis* and associated risk factors among African children.

**Methods:**

We searched online databases (PubMed, Scopus, and Web of Science) as well as the Google Scholar search engine for studies measured the prevalence of *G. duodenalis* among African children, published between 1 January 2000 and 15 March 2022. Due to high heterogeneity among the included studies, a random-effects meta-analysis model was employed to estimate pooled prevalence and 95% confidence intervals (CI).

**Results:**

A total of 114 articles from 29 African countries met the inclusion criteria. The pooled prevalence of *G. duodenalis* infection among African children was estimated as 18.3% (95% CI: 16.5–20.2). The highest and lowest pooled prevalence of *G. duodenalis* infection were estimated in Niger and Cameroon as 65.1% (55–75.2) and 0.08% (0.02–1.05), respectively. Considering the type of study population, the highest prevalence was related to, iron-deficient children 65.2% (61.3–69.1), handicapped children 30.4% (18.3–42.4), HIV infected children 25.7% (11.2–40.2) and displaced children 20.2% (16.5–23.9).

**Conclusions:**

Giardiasis is common among African children, hence, prevention and control scheme of this protozoan in children should be considered by health officials and health policymakers, especially in African countries where prevalence is highest.

## 1 Background

*Giardia duodenalis* (*G. duodenalis*) is a protozoan flagellate infecting the intestinal tract of human and animals with a worldwide distribution (Adam Rodney, 2021; Dixon, 2021; Taghipour et al., 2022b). The fecal–oral route, including direct (i.e., person-to-person, animal-to-animal or zoonotic) or indirect (i.e., waterborne or foodborne) are the main transmission mode of *G. duodenalis* by consumption of infective cyst (Adam Rodney, 2021; Dixon, 2021; Zahedi et al., 2020). Spreading of *G. duodenalis* in different communities is related to sanitation level, Human Development Index (HDI), income level, and drinking water quality (Horton et al., 2019). Therefore, the occurrence of *G. duodenalis* is much lower in developed countries than in less-developed countries (Gilman et al., 1985; Muhsen and Levine, 2012). Some populations, including children, pregnant women, and immunocompromised people are at higher risk of *G. duodenalis* infection (Adam Rodney, 2021; Jeske et al., 2022; Rasti et al., 2017; Taghipour et al., 2021).

The common clinical symptoms/signs of giardiasis include fatty stools (steatorrhea), nausea, vomiting, abdominal discomfort, abdominal bloating, cramps, malabsorption, and weight loss (Sahagun et al., 2008; Taghipour et al., 2022a). Among clinical manifestations, diarrhea is the cause of mortality of about 480,000 young children worldwide, responsible for 9% of all deaths among children under 5 years of age in 2019 (https://data.unicef.org/topic/child-health/diarrhoeal-disease/.). Based on the World Health Organization (WHO) reports in 2010, giardiasis is assessed to cause ∼28.2 million cases of diarrhea (Havelaar et al., 2015; Organization, W.H, 2015). Moreover, chronic giardiasis is related with food allergies (Di Prisco et al., 1998), irritable bowel syndrome (IBS) (Abedi et al., 2021), chronic fatigue syndrome (Hanevik et al., 2017), arthritis (Painter et al., 2017), as well as growth deficiency in children (Dougherty and Bartelt, 2022).

Children are more exposed to different environmental sources (e.g., playing with soil) and also have immature immune systems to fight infections (Moya et al., 2004; Mussi-Pinhata and Nascimento, 2001). Therefore, children have a higher probability of contracting infectious agents. Determination of the epidemiological patterns of *G. duodenalis* infection is necessary to design future control programs and preventive measures to reduce the incidence of the infection. To address this gap, we designed a systematic review and meta-analysis to assess the prevalence of *G. duodenalis* and associated risk factors in African children.

## 2 Materials and methods

### 2.1 Information sources and systematic search

The present systematic review and meta-analysis was conducted based on the Preferred Reporting

Items for Systematic Reviews and Meta-analyses (PRISMA) protocol (Moher et al., 2015). Published articles on the prevalence of *G. duodenalis* in African children was gathered through three international databases (i.e. PubMed, Scopus, and Web of Science) and Google Scholar search engine between 1 January 2000 and 15 March 2022. The search process was accomplished using Medical Subject Headings (MeSH) terms alone or in combination: (“Intestinal protozoa” OR “*Giardia*” OR “Giardiasis”) AND (“Prevalence” OR “Epidemiology”) AND (“Children”). Moreover, the references list of all selected articles was hand-searched to find other relevant articles or their citations by searching in Google Scholar.

### 2.2 Inclusion criteria, study selection and data extraction

To assess the article eligibility based on determined inclusion criteria, all papers were reviewed by two independent reviewers and possible contradictions among studies were removed by discussion and consensus. The inclusion criteria for this systematic review were as follows: (1) full-texts or abstracts published in English from Africa continent; (2) peer-reviewed original research papers or short reports; (3) cross-sectional studies that estimated the prevalence of *Giardia* in children population (≤18 years); (4) utilizing fecal microscopy, coproantigen or molecular diagnostic methods; (5) reports with information on the total sample size and positive samples; and (6) published online from 1 January 2000 and 15 March 2022. Those papers without full-text accessibility or papers that did not meet the above criteria were excluded. Next, the desired data were gathered precisely using a data extraction form including, the first author's last name, the year the study was conducted and the publication year, countries, provinces and cities, types of method used, total sample sizes, number of positive samples, types of children, gender and age of children.

### 2.3 Study quality assessment

The Joanna Briggs Institute (JBI) checklist was applied for the risk of bias (internal validity) assessment of the included articles (Porritt et al., 2014). This checklist comprises ten questions with four options including Yes, No, Unclear, and Not applicable. Summary, a study can be awarded a maximum of one star for each numbered item. The papers with a total score of 4–6 and 7–10 points were specified as the moderate and high quality, respectively. Based on the obtained score, the authors have decided to include (4–10 points) and exclude (≤3 points) the papers.

### 2.4 Meta-analysis

For each included study, the point estimates and their respective 95% confidence intervals (CI) using a random effect model (REM) were calculated. The REM allows for a distribution of true effect sizes between articles. To visualize possible heterogeneity among included studies the forest plot analysis was used. The heterogeneity index among the included studies was defined using the *I*^*2*^ index and Tau squared to reveal the variation in study outcomes between individual studies (Barati et al., 2022; Maleki et al., 2021). The univariate and multivariable meta-regression analysis was used to estimate the effects of probable factors in heterogeneity (Barati et al., 2022). To investigate the effect of each study on the pooled estimation of prevalence, the sensitivity analysis method was used by removing studies one by one. The robustness of each model was evaluated and finally, the most favorable model was chosen.

Using sub-group analyses, the pooled prevalence of *Giardia* infection was estimated according to countries, types of diagnostic methods, types of children, and periods of studies. An odds ratio (OR) (and the corresponding 95% (CI)) was calculated for each study to assess the association between *Giardia* spp. prevalence and risk factors such as sex (male and female) and place of living (rural and urban).

The publication bias was evaluated with Egger's regression test and interpretation of the funnel plot in this meta-analysis and the trim-and-fill method was used to estimate the number of censored studies and correct the overall estimate (Barati et al., 2022).

Moreover, due to different sensitivities and specificities of diagnostic methods, we assumed that our results would be “apparent” prevalence rates, and did not represent true prevalence rates. The prevalence of *G. duodenalis* in children in different countries from Africa was demonstrated as a world map using ArcGIS 10.3 software (https://www.arcgis.com). Source of vector map is from ESRI (www.esri.com/en-us/home). This meta-analysis was conducted with the Stata version 16 software and the trial version of Comprehensive Meta-Analysis software vs. 3. A *P*-value of <0.05 was considered significant.

## 3 Result

### 3.1 Characteristics of the eligible studies

**Fig. 1:**
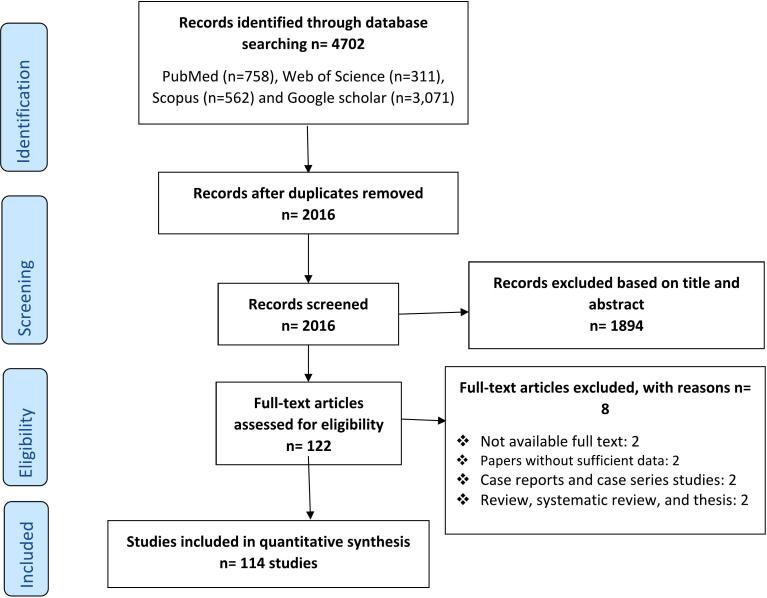
PRISMA flow diagram describing included/excluded studies.

A flowchart depicting the identification process of qualifying studies is presented in Fig. 1. In brief, the systematic search identified 4702 potentially relevant articles. After removing duplicates and/or non-eligible papers, 114 articles from 29 countries across Africa met the inclusion criteria in the systematic review and meta-analysis. The countries with the highest number of studies were Ethiopia (17.54%; 20/114 studies) and Egypt (16.66%; 19/114 studies). The main characteristics of each study are shown in the Supplementary Table 1. The results of quality assessment according to JBI with references for eligible studies are depicted in Supplementary Table 1. The included articles in the present meta-analysis showed an acceptable quality.

### 3.2 The pooled prevalence of G. Duodenalis in children

A total of 63,165 children were evaluated in 114 studies regarding the prevalence of *G. duodenalis* in African children, of which 10,202 were diagnosed as infected based on diagnostic criteria. After performing sensitivity analysis by removing one-by-one studies and selecting the robustness model, the overall prevalence of *G. duodenalis* infection in African children was estimated at 18.3% (95% CI,16.5–20.2) using the REM (Supplementary Fig. 1).

According to the heterogeneous assessment indicators between studies (Q value = 11,448.44, d.f. = 113, *p* < 0.0001, *I*^2^ = 99.0%, Tau squared = 0.0097), it was shown that there is a high heterogeneity between the studies included in this meta-analysis. In the next step, multivariable and univariate meta-regression models were used to find the origin of heterogeneity (Table 1). Multivariable meta-regression analysis showed that the type of population studied in children it may be the source of heterogeneity (*p* = 0.037), however, this analysis did not show significant heterogeneity in the quality of the studies included in the meta-analysis, study location, sample size, diagnostic methods, and age groups (*P* > 0.05) (Table 1). In addition, the univariate meta-regression model showed that the location of the study (country) (*p* = 0.006) and the type of population under study (*p* = 0.004) may be the causes of heterogeneity (Table 1).

**Table 1:** Result of multivariable and univariate meta-regression model to identify possible sources of heterogeneity.

The probable source of heterogeneity	Multivariable			Univariate		
	Coefficient (95%CI)	P-value	Adj R-squared	Coefficient (95%CI)	P-value	Adj R-squared
Years of publication	−0.00101(−0.00716 to 0.00514)	0.745	9.76% (Multivariable)	−0.00040(−0.00634 to 0.00553)	0.893	−0.91%
Risk of bias	−0.02642(−0.06240 to 0.00955)	0.148		−0.03138(−0.06370 to 0.00093)	0.057	2.42%
Country	0.00705(−0.00410 to 0.05515)	0.117		0.01821 (0.01042 to 0.0483)	0.006⁎	12.18%
Sample size	−0.00001(−0.00005 to 0.00003)	0.556		−0.00002(−0.00006 to 0.00001)	0.248	0.32%
Detection method	−0.01604(−0.05734 to 0.02526)	0.443		−0.01613(−0.05660 to 0.02433)	0.431	−0.35%
Population under study	−0.00365(−0.01470 to-0.00738)	0.037⁎		−0.00318(−0.01376 to −0.00739)	0.004⁎	10.14%
Age group	−0.00007(−0.04987 to 0.04973)	0.998		−0.00267(−0.05113 to 0.04579)	0.913	−0.91%

⁎ Statistically significant (P-value≤0.05)

**Fig. 2:**
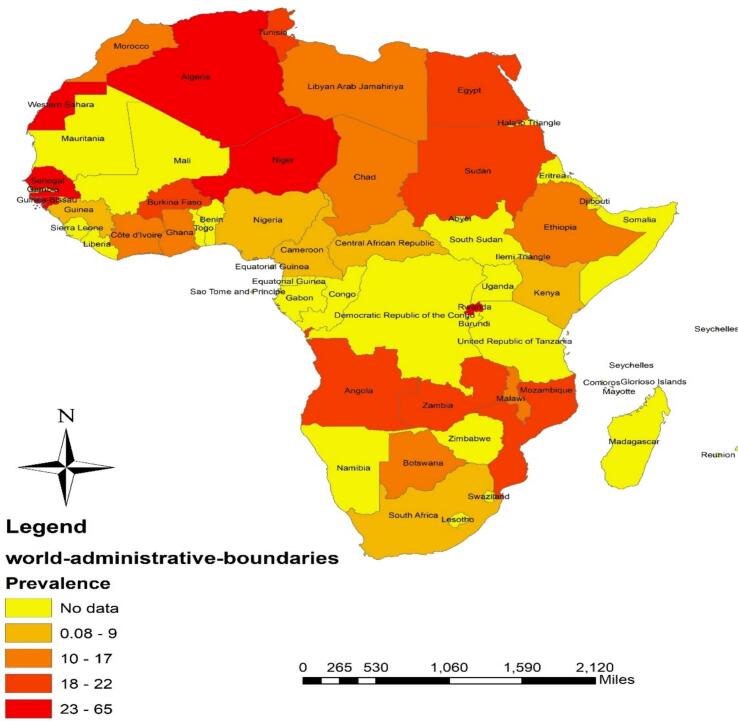
Prevalence of *G. duodenalis* in children on the African continent.

The subgroup meta-analysis using the REM was used to estimate the overall prevalence in groups including country, type of study population, year of publication, detection method, and age group (Table 2). According to the location of the study, the highest prevalence of *G. duodenalis* was in African children in Niger 65.1% (55–75.2), Algeria 54.5% (1.04–98.07), Senegal 45.2% (40.4–50.1), and Guinea-Bissau 40.6% (30.9–50.2), respectively. On the other hand, the lowest prevalence was related to Cameroon 0.08% (0.02–1.05), and the Central African Republic 0.09% (0.001–1.09) (Table 2 and Fig. 2).

**Table 2:** The overall Prevalence *G. duodenalis* in African children.

		No. studies	No. examined	No. positive	Prevalence (95%CI)	Heterogeneity			
						χ^2^	P-value	*I*^2^ (%)	Tau-squared
Country	Algeria	2	294	111	54.5% (1.04–98.07)	82.97	<0.001	98.8%	0.1451
	Angola	1	328	66	20.1% (15.8–24.5)	NA	NA	NA	NA
	Botswana	1	200	33	16.5% (11.4–21.6)	NA	NA	NA	NA
	Burkina Faso	2	694	143	19.6% (3.02–36)	32.94	<0.001	97.0%	0.0136
	Cameroon	1	831	7	0.08% (0.02–1.05)	NA	NA	NA	NA
	Central African Republic	1	333	3	0.09% (0.001–1.09)	NA	NA	NA	NA
	Chad	1	200	21	10.5% (6.03–14.7)	NA	NA	NA	NA
	Côte d'Ivoire	2	1704	259	17.1% (10.3–23.9)	7.75	0.005	87.1%	0.0021
	Egypt	19	7592	1642	22.1% (14.6–29.6)	1746.61	<0.001	99.0%	0.0270
	Ethiopia	20	6493	1063	13.9% (9.03–18.5)	947.43	<0.001	98.0%	0.0107
	Ghana	4	5073	866	16.4% (6.07–26.1)	276.07	<0.001	98.9%	0.0097
	Guinea	1	392	20	5.01% (2.09–7.03)	NA	NA	NA	NA
	Guinea-Bissau	3	588	224	40.6% (30.9–50.2)	7.54	0.023	73.5%	0.0051
	Kenya	5	5924	357	6.04% (3.09–9.0)	70.63	<0.001	94.3%	0.0008
	Libya	7	3273	387	11.6% (5.01–18)	357.73	<0.001	98.3%	0.0072
	Malawi	2	228	33	17.2% (4.07–29.7)	2.93	0.087	65.8%	0.0058
	Morocco	3	1419	200	14% (11.1–16.8)	4.61	0.100	56.6%	0.0004
	Mozambique	6	7028	1813	19.8% (9.08–29.9)	499.67	<0.001	99.0%	0.0155
	Nigeria	10	12,719	757	9.06% (6.09–12.2)	607.48	<0.001	98.5%	0.0016
	Niger	1	86	56	65.1% (55–75.2)	NA	NA	NA	NA
	Rwanda	4	1985	855	38.7% (10.5–66.9)	740.96	<0.001	99.6%	0.0822
	Sudan	8	2191	475	21.7% (14.9–28.4)	114.13	<0.001	93.9%	0.0086
	Tanzania	3	1524	295	17.8% (5.03–41.1)	286.46	<0.001	99.3%	0.0413
	Zambia	2	1115	261	19.5% (0.09–38.1)	67.15	<0.001	98.5%	0.0177
	Sahrawi	1	120	41	34.2% (25.7–42.7)	NA	NA	NA	NA
	São Tomé and Prín	1	134	10	7.05% (3–11.9)	NA	NA	NA	NA
	Several countries in Africa	1	135	7	5.02% (1.04–8.09)	NA	NA	NA	NA
	South Africa	1	162	16	9.09% (5.03–14.5)	NA	NA	NA	NA
	Senegal	1	400	181	45.2% (40.4–50.1)	NA	NA	NA	NA
Type of study population	Children	51	27,701	5116	19.3% (16.2–22.4)	5085.47	<0.001	99.0%	0.0124
	Children with diarrhea	16	6070	1038	17% (12–21.9)	516.57	<0.001	97.1%	0.0095
	Displaced children	1	450	91	20.2% (16.5–23.9)	NA	NA	NA	NA
	Handicapped children	1	56	17	30.4% (18.3–42.4)	NA	NA	NA	NA
	HIV Infected Children	1	35	9	25.7% (11.2–40.2)	NA	NA	NA	NA
	Iron-deficient children	1	575	375	65.2% (61.3–69.1)	NA	NA	NA	NA
	Preschool children	4	1205	143	11.3% (7.08–14.7)	10.85	0.013	72.4%	0.0009
	Primary school children	11	8105	790	13% (8.09–17)	786.69	<0.001	98.7%	0.0044
	School Children	27	19,391	2712	18.5% (14.6–22.9)	3443.14	<0.001	99.2%	0.0107
	Children with allergy	1	27	3	7.04% (2.05–17.03)	NA	NA	NA	NA
Diagnostic method	ELISA	1	274	25	9.01% (5.07–12.5)	NA	NA	NA	NA
	Microscopic examination	94	52,103	7363	16.3% (14.5–18.1)	8430.46	<0.001	98.9%	0.0079
	Molecular examination	6	1558	381	22.7% (8.03–37.1)	384.88	<0.001	98.7%	0.0311
	Real-time PCR	10	7296	2098	36.6% (24.9–48.4)	1412.18	<0.001	99.4%	0.0349
	Immunoassays	1	983	25	9.07% (7.08–11.5)	NA	NA	NA	NA
	Immunofluorescent microscopy	1	120	41	34.2% (25.7–42.7)	NA	NA	NA	NA
	Rapid immunochromatographic test	1	831	199	23.9% (21–26.8)	NA	NA	NA	NA
Year	2000–2005	10	11,353	876	17.4% (11.8–23.0)	1696.47	<0.001	99.5%	0.0078
	2006–2011	23	10,834	2511	22.1% (17.0–27.33)	1175.61	<0.001	98.1%	0.0153
	2012–2017	52	25,865	3985	17.1% (14.5–19.7)	4633.68	<0.001	98.9%	0.0085
	2018–2022	29	15,113	2830	18% (13.7–22.2)	2318.93	<0.001	98.8%	0.0131
Age group	<5 years	47	24,378	4382	17.9% (14.7–21.1)	4779.66	<0.001	99.0%	0.0120
	5–12 years	55	25,572	4333	19.5% (16.6–22.5)	5576.79	<0.001	99.0%	0.0120
	12–19 years	12	13,215	1487	14.9% (10.4–19.4)	1035.52	<0.001	98.9%	0.0059

NA: Not applicable, ELISA: enzyme-linked immunosorbent assay; PCR: polymerase chain reaction.

Based on the type of studied groups of children, the highest prevalence was related to, iron-deficient children 65.2% (61.3–69.1), handicapped children 30.4% (18.3–42.4), HIV infected children 25.7% (11.2–40.2) and displaced children 20.2% (16.5–23.9), respectively (Table 2). Based on the age groups of the studied children, the prevalence of *G. duodenalis* in African children was estimated at under 5 years 17.9% (14.7–21.1), 5–12 years 19.5% (16.6–22.5) and 12–19 years 14.9% (10.4–19.4), respectively.

The prevalence of *G. duodenalis* in children on the African continent was estimated at 17.4% (11.8–23.0) in 2000–2005, 22.1% (17.0–27.33) in 2006–2011, 17.1% (14.5–19.7) in 2012–2017, and 18% (13.7–22.2) in 2018–2022 (Table 2).

### 3.3 Risk factors

**Fig. 3:**
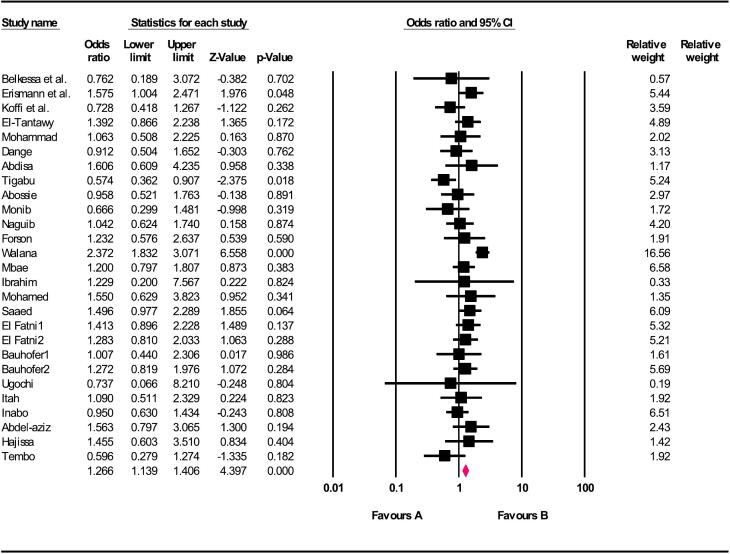
Relationship between gender and *G. duodenalis* in children of Africa.

**Fig. 4:**
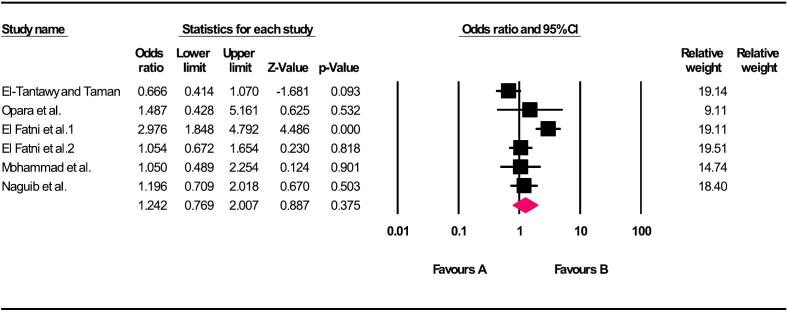
Relationship between the living area and *G. duodenalis* in children of Africa.

Based on children's sex, boys had a higher risk for *G. duodenalis* than girls (OR = 1.26; 95% CI: 1.13–1.40, *p* < 0.001) (Q value = 52.84, d.f. = 26, *p* = 0.001, *I*^2^ = 50.79%, Tau squared = 0.082), (Fig. 3). Additionally, living in a rural area compared to an urban area increased the chance of contracting *G. duodenalis* by 1.24 times, but this increase was not statistically significant (OR = 1.24;95% CI:0.76–2.00, *P* = 0.375) (Q statistic = 20.35, d.f. = 5, p = 0.001, *I*^2^ = 75.43%, Tau squared = 0.254) (Fig. 4).

### 3.4 Publication bias

**Fig. 5:**
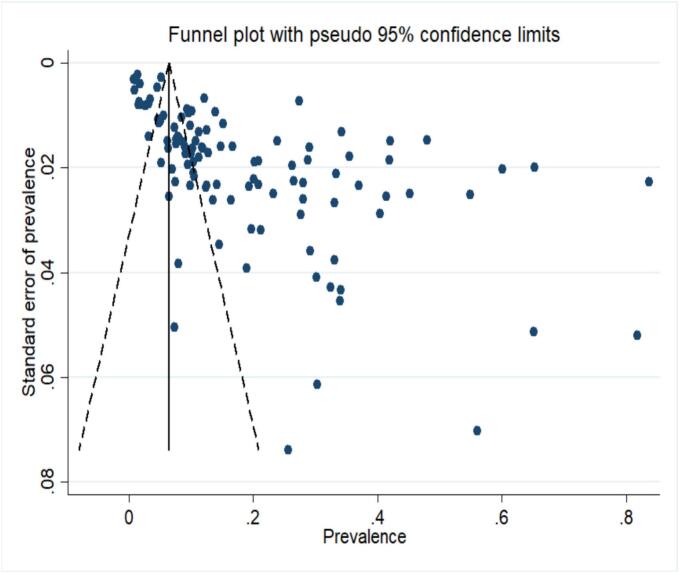
Funnel plot with 95% confidence interval for assessing the publication bias.

Egger's regression test and asymmetry in the funnel plot displays that there is significant publication bias for included studies in this meta-analysis (bias = 10.018, 95%CI: 7 7.941–12.096, *P* < 0.001) (Fig. 5). Therefore, the trim-and-fill model using non-parametric methods was used for correction the meta-analysis. This model showed that 54 hypothetical studies about the prevalence of *G. duodenalis* in African children were censored in this meta-analysis. Consequently, the pooled prevalence of *G. duodenalis* corrected by the REM based on the trim-and-fill model was estimated to be 4.09% (95%CI: 2.09–7.0).

## 4 Discussion

This is the first study to estimate the prevalence of *G. duodenalis* among African children. The prevalence obtained for *G. duodenalis* in the meta-analysis were relatively high, especially in studies that have used the Real-time PCR method (Table 2). Conventionally, microscopy detection method using staining procedures is considered as the gold standard method for the detection of cysts and/or trophozoites of *G. duodenalis* (Soares and Tasca, 2016). However, molecular methods are preferred for conducting research activities because they have higher sensitivity and specificity and the interpretation of results is easier (David and Ap, 2006; Elmi et al., 2017). Accordingly, the pooled prevalence provided by molecular methods could be closer to the true prevalence. For a deeper understanding of this issue, it is necessary for researchers to use molecular methods in addition to microscopy methods.

By considering the prevalence of *G. duodenalis* in different African countries, the highest infection rates were found in Niger (one study with pooled prevalence of 65.1%) and Algeria (two studies with pooled prevalence of 54.5%). By contrast, infection rates in Cameroon (one study with pooled prevalence of 0.08%) and Central African Republic (one study with pooled prevalence of 0.09%) countries were low. Several environmental and sociodemographic parameters are complicated in the different prevalence rates obtained, including climatic condition, parasite control measures, *HDI*, and the use of diverse diagnostic methods in different regions (Barasa et al., 2024; Kalavani et al., 2024; Koh et al., 2024; Weitzel et al., 2024). In addition to all these factors, it is necessary to conduct more studies in African countries and some countries that have not done any research on this issue should consider it; finally, a deeper understanding of its prevalence in children in different parts of Africa can be obtained.

Considering the year of publication (Table 2), the prevalences in different time periods from 2000 to 2022 were almost similar. Therefore, the results of this meta-analysis study, especially the pooled prevalence rates based on the year of publication, should be interpreted with caution. Hence, factors such as the number of published articles and the sample size of studies each year may play a role in causing heterogeneity.

Living in a rural area compared to an urban area, and boys compared to girls increased the chance of contracting *G. duodenalis* by 1.24 and 1.26 times, respectively, which might be explained by lower personal hygiene scores and more contact with *G. duodenalis* cysts-contaminated water and vegetables.

The present study has a number of limitations. First, despite our comprehensive search, there was a paucity or absence of data for a number of countries, and many of the available studies had limited sample sizes and a lack of data on socio-demographic and/or risk factors. Moreover, in some countries only one or two eligible studies was identified, which could compromise somewhat the interpretation of present estimates. Second, studies included were undertaken during different time periods, with an absence of recent data for some countries, limiting the accuracy of inter-regional comparisons. Third, there was a high heterogeneity in this meta-analysis. Although we investigated its possible source by performing meta-regression analysis.

## 5 Conclusions

In summary, prevention and control scheme of *G. duodenalis* in children should receive greater attention by health officials and health policymakers, especially in African countries where prevalence is highest. Also, we recommend that periodic screenings for *G. duodenalis* in such countries should be incorporated into the routine clinical care of iron-deficient children, handicapped children, HIV infected children, and displaced children.

## Funding

This study was supported by Zoonoses Research Center, Jahrom University of Medical Sciences, Jahrom, Iran.

## Ethics approval and consent to participate

This study was approved by Jahrom University of Medical Sciences Ethics Committee (ethical approval ID: IR.JUMS.REC.1401.018).

## Consent for publication

Not applicable.

## CRediT authorship contribution statement

**Sara Matin:** Supervision, Methodology, Conceptualization. **Vahid Rahmanian:** Software, Formal analysis. **Ahmad Meshkin:** Investigation. **Bahareh Bahadori Mazidi:** Methodology. **Ali Taghipour:** Writing – review & editing, Writing – original draft, Supervision, Methodology, Conceptualization. **Amir Abdoli:** Writing – original draft.

## Declaration of competing interest

The authors declare that they have no known competing financial interests or personal relationships that could have appeared to influence the work reported in this paper.

## Data Availability

All data during study are included in this manuscript and Supplementary Files.

## Appendix A Supplementary data

**Supplementary Table 1.** Main characteristics of all eligible studies reporting prevalence of *Giardia duodenalis* in African children. Image 1

Supplementary Figure 1. Results of sensitivity analysis based on individual study removal for estimating the prevalence of *G. duodenalis* in children. Image 2
