# Supplementary material for: Prevalence of Giardia duodenalis among African children: A systematic review and meta-analysis
Source: Parasite Epidemiol Control. 2024 Jul 4;26:e00365. doi: 10.1016/j.parepi.2024.e00365 (PMC11277988; doi:10.1016/j.parepi.2024.e00365)
Supplement: Supplementary file 1 — Supplementary Table 1. Main characteristics of all eligible studies reporting prevalence of Giardia duodenalis in African children. [file mmc1.docx]

| **Supplementary Table 1.** Main characteristics of all eligible studies reporting prevalence of *Giardia duodenalis* in African children. | | | | | | | | | |
| --- | --- | --- | --- | --- | --- | --- | --- | --- | --- |
| **First author** | **Publication Year** | **Type of population** | **Age range or Mean age** | **Diagnostic method** | **Country** | **Sample size** | **Infected** | **QA** | **Reference** |
| Asfaw and Goitom | 2000 | Children | Under 5 years | Microscopic examination | Ethiopia | 330 | 25 | 10 | [1] |
| Ibrahim et al. | 2000 | Primary school children | 5-14 years | Microscopic examination | Nigeria | 2762 | 38 | 10 | [2] |
| Okaka et al. | 2000 | School children 6-20 years | 6-20 years | Microscopic examination | Nigeria | 6430 | 339 | 10 | [3] |
| Diouf et al. | 2000 | Children aged ≤5 years | ≤5 years | Microscopic examination | Senegal | 400 | 181 | 10 | [4] |
| Gascon et al. | 2000 | Children with diarrhea | Under 5 years | Microscopic examination | Tanzania | 103 | 15 | 8 | [5] |
| Ukpai and Ugwa | 2003 | Primary school children | 5-16 years | Microscopic examination | Nigeria | 300 | 5 | 9 | [6] |
| Rice et al. | 2003 | Children | 0-17 years | Microscopic examination | Several countries from Africa | 135 | 7 | 8 | [7] |
| Legesse and Erko | 2004 | School children | 6->15 years | Microscopic examination | Ethiopia | 259 | 16 | 9 | [8] |
| Megahed et al. | 2005 | School children | NR | Microscopic examination | Egypt | 264 | 221 | 9 | [9] |
| Itah et al. | 2005 | Children | 3-17 years | Microscopic examination | Nigeria | 370 | 29 | 10 | [10] |
| Adamu et al. | 2006 | Children with diarrhea | Under 5 years | Microscopic examination | Ethiopia | 222 | 14 | 9 | [11] |
| Aida et al. | 2006 | School children | 7-14 years | Microscopic examination | Libya | 601 | 173 | 10 | [12] |
| El-Masry et al. | 2007 | School children | 6-16 years | Microscopic examination | Egypt | 960 | 146 | 10 | [13] |
| Ben Musa | 2007 | School children | 5-18 years | Microscopic examination | Libya | 50 | 4 | 7 | [14] |
| Kasssem et al. | 2007 | Children and neonatus | NR | Microscopic examination | Libya | 350 | 36 | 10 | [15] |
| El-Sherbini et al. | 2008 | Handicapped children | 5-16 years | Microscopic examination | Egypt | 56 | 17 | 7 | [16] |
| Faiza et al. | 2009 | Preschool children | 2-5 years | Microscopic examination | Egypt | 300 | 23 | 10 | [17] |
| Atia | 2009 | Children | NR | Microscopic examination | Egypt | 1154 | 554 | 10 | [18] |
| Worku et al. | 2009 | School children | 6-14 years | Microscopic examination | Ethiopia | 322 | 29 | 10 | [19] |
| Nyamngee et al. | 2009 | Children from GUMA refugee camp | 0-19 years | Microscopic examination | Nigeria | 292 | 118 | 9 | [20] |
| Ogbolu et al. | 2009 | Primary school children | 2-16 years | Microscopic examination | Nigeria | 394 | 47 | 10 | [21] |
| Lalle et al. | 2009 | Children | 8-13 years | Immunofluorescent microscopy | Sahrawi | 120 | 41 | 9 | [22] |
| Abdel aziz et al. | 2009 | School children | NR | Microscopic examination | Sudan | 157 | 31 | 8 | [23] |
| Ouattara et al. | 2010 | School children | NR | Microscopic examination | Côte d'Ivoire | 1398 | 195 | 10 | [24] |
| Nyantekyi et al. | 2010 | Children | ≤ 5 years | Microscopic examination | Ethiopia | 288 | 67 | 9 | [25] |
| Tigabu et al. | 2010 | Children | 2 months-14 years | Microscopic examination | Ethiopia | 384 | 102 | 10 | [26] |
| Abdel-aziz et al. | 2010 | School children | NR | Microscopic examination | Sudan | 157 | 52 | 8 | [27] |
| Nitiema et al. | 2011 | Children with diarrhea | 0->24 months | Microscopic examination | Burkina Faso | 309 | 35 | 10 | [28] |
| Ayalew et al. | 2011 | School children | NR | Microscopic examination | Ethiopia | 704 | 295 | 10 | [29] |
| Nkrumah and Nguah | 2011 | Children | Less than 18 years | Microscopic examination | Ghana | 1080 | 101 | 10 | [30] |
| Thiongo et al. | 2011 | Children | Up to 5 years | Microscopic examination | Kenya | 376 | 48 | 10 | [31] |
| Inabo | 2011 | Primary school children | 0-12 years | Microscopic examination | Nigeria | 374 | 155 | 10 | [32] |
| Siwila et al. | 2011 | Children | NR | Microscopic examination | Zambia | 786 | 228 | 10 | [33] |
| Abdel-Hafeez et al. | 2012 | Children | NR | Microscopic examination | Egypt | 450 | 33 | 10 | [34] |
| Mohammad et al. | 2012 | School children | 4-19 years | Microscopic examination | Egypt | 530 | 30 | 10 | [35] |
| Krumkamp et al. | 2012 | Children | <15 years | Microscopic examination | Ghana | 1293 | 443 | 10 | [36] |
| Ferreira et al. | 2012 | Children | NR | Molecular examination | Guinea-Bissau | 50 | 28 | 7 | [37] |
| Opara et al. | 2012 | Primary school children | 2.9-14 years | Microscopic examination | Nigeria | 405 | 11 | 10 | [38] |
| Ignatius et al. | 2012 | Children | < 5 years | Microscopic examination | Rwanda | 583 | 350 | 10 | [39] |
| Mohamed et al. | 2012 | Displaced children | 6 months-13 years | Microscopic examination | Sudan | 450 | 91 | 10 | [40] |
| Sadek et al. | 2013 | Children | 5-12 years | Microscopic examination | Egypt | 161 | 47 | 8 | [41] |
| Unasho | 2013 | Children | 5 months-5 years | Microscopic examination | Ethiopia | 406 | 11 | 10 | [42] |
| Centeno-Lima et al. | 2013 | Children | Under five years | Microscopic examination | Guinea-Bissau | 109 | 37 | 8 | [43] |
| Mbae et al. | 2013 | Children | 0-5 years | Microscopic examination | Kenya | 2112 | 98 | 10 | [44] |
| Al-Mubrook et al. | 2013 | Children | NR | Microscopic examination | Libya | 501 | 16 | 10 | [45] |
| Nxasana et al. | 2013 | Primary school children | 4-11 years | Microscopic examination | South Africa | 162 | 16 | 8 | [46] |
| Fusi-Ngwa et al. | 2014 | Children | 6 months-18 years | Microscopic examination | Cameroon | 831 | 7 | 10 | [47] |
| Koffi et al. | 2014 | Children with diarrhea | Under 5 years | Molecular examination | Côte d’Ivoire | 306 | 64 | 10 | [48] |
| Helmy et al. | 2014 | Children with diarrhea | Below the age of 10 years | Real time PCR assay | Egypt | 165 | 35 | 8 | [49] |
| El-Tantawy and Taman | 2014 | Children with diarrhea | 3-12 years | Microscopic examination | Egypt | 311 | 103 | 8 | [50] |
| Hegazy et al. | 2014 | Preschool children | 2-6 years | Microscopic examination | Egypt | 500 | 74 | 10 | [51] |
| Firdu et al. | 2014 | Children | ≤4-13 years | Microscopic examination | Ethiopia | 230 | 22 | 9 | [52] |
| Asemahagn | 2014 | School children |  | Microscopic examination | Ethiopia | 358 | 29 | 10 | [53] |
| Abossie and Seid | 2014 | School children | 5-15 years | Microscopic examination | Ethiopia | 400 | 47 | 10 | [54] |
| Walana et al. | 2014 | Primary school children | 5-12 years | Microscopic examination | Ghana | 2400 | 292 | 10 | [55] |
| Pavlinac et al. | 2014 | Children with HIV-Infected and HIV-Uninfected | NR | Microscopic examination | Kenya | 1076 | 109 | 10 | [56] |
| Messaad et al. | 2014 | Children | 0-15 years | Microscopic examination | Morocco | 200 | 25 | 9 | [57] |
| El Fatni et al. | 2014 | Children | NR | Microscopic examination | Morocco | 546 | 91 | 10 | [58] |
| El Fatni et al. | 2014 | Children | 5-14 years | Microscopic examination | Morocco | 673 | 84 | 10 | [59] |
| Fonseca et al. | 2014 | Children | NR | Microscopic examination | Mozambique | 93 | 6 | 7 | [60] |
| Ignatius et al. | 2014 | Children | NR | Microscopic examination | Rwanda | 474 | 99 | 10 | [61] |
| Danquah et al. | 2014 | Iron-deficient children | Under 5 years | Microscopic examination | Rwanda | 575 | 375 | 10 | [62] |
| Lobo et al. | 2014 | Children | NR | Molecular examination | São Tomé and Príncipe | 134 | 10 | 8 | [63] |
| Gabbad and Elawad | 2014 | Primary school children | NR | Microscopic examination | Sudan | 500 | 167 | 10 | [64] |
| Oliviera et al. | 2015 | School children | 5-12 years | Microscopic examination | Angola | 328 | 66 | 9 | [65] |
| Al-Ghwass et al. | 2015 | Children with allergy | 2-13 years | Microscopic examination | Egypt | 27 | 2 | 7 | [66] |
| Mulatu et al. | 2015 | Children with diarrhea | <5 years | Microscopic examination | Ethiopia | 158 | 11 | 8 | [67] |
| Aleka et al. | 2015 | Children under 5 years | 5 years and below | Microscopic examination | Ethiopia | 277 | 5 | 9 | [68] |
| Yones et al. | 2015 | Children | 1-6 years | Microscopic examination | Eygpt | 300 | 84 | 9 | [69] |
| Ugochi et al. | 2015 | Primary School Children | 5-16 years | Microscopic examination | Nigeria | 337 | 3 | 10 | [70] |
| Saeed et al. | 2015 | Children with diarrhea | <5 years | Microscopic/PCR | Sudan | 437 | 47 | 9 | [71] |
| Ngosso et al. | 2015 | Children with either acute or chronic diarrhea | <5 years | PCR | Tanzania | 720 | 256 | 10 | [72] |
| Tellevik et al. | 2015 | Children with and without Diarrhea | <2 years | Real time PCR assay | Tanzania | 701 | 24 | 10 | [73] |
| Erismann et al. | 2016 | School Children | 8-14 years | Microscopic examination | Burkina Faso | 385 | 108 | 10 | [74] |
| Breurec et al. | 2016 | Hospitalized Children | <5 years | Microscopic examination | Central African Republic | 333 | 3 | 10 | [75] |
| Lucio et al. | 2016 | School Children | 15-Jun | Real time PCR | Ethiopia | 393 | 216 | 10 | [76] |
| Madbouly et al. | 2016 | Diarrheal Children | 0-12 years | Microscopic/PCR/ELISA | Eygpt | 100 | 19 | 7 | [77] |
| Monib et al. | 2016 | Children attending Assiut university child's hospital | 4m-5 years | Microscopic examination | Eygpt | 260 | 27 | 9 | [78] |
| Mbae et al. | 2016 | Children | 5 years and below | Microscopic examination | Kenya | 2112 | 98 | 10 | [79] |
| Aiemjoy et al. | 2017 | Preschool aged Childern | 0-5 years | Microscopic examination | Ethiopia | 212 | 22 | 9 | [80] |
| El-Bardy et al. | 2017 | Diarrheal Children | 5-12 years | Microscopic examination | Eygpt | 126 | 38 | 8 | [81] |
| Forson et al. | 2017 | School Children | 2-9 years | Microscopic examination | Ghana | 300 | 30 | 10 | [82] |
| Esiet and Edet | 2017 | Children in public and private schools | 6-13 years | Microscopic examination | Nigeria | 1055 | 12 | 10 | [83] |
| Sidding et al. | 2017 | Primary School Children | 5-14 years | Microscopic examination | Sudan | 120 | 39 | 8 | [84] |
| Abd-Elhafiz et al. | 2017 | Children | 6-18 years | Microscopic examination | Sudan | 200 | 25 | 9 | [85] |
| Feleke et al. | 2018 | Under 5 years children | <5 years | Microscopic examination | Ethiopia | 225 | 32 | 9 | [86] |
| Naguib et al. | 2018 | Children | 2-8 years | Real time PCR assay | Eygpt | 585 | 66 | 10 | [87] |
| Ibrahim et al. | 2018 | Children | 1m-14 years | Microscopic examination | Libya | 156 | 5 | 8 | [88] |
| Huibers et al. | 2018 | HIV Infected Children | 18m-18 years | Real time PCR assay | Malawi | 35 | 9 | 7 | [89] |
| Mekonnen and Ekubagewargies | 2019 | Children under 5 years | <5 years | Microscopic examination | Ethiopia | 310 | 31 | 10 | [90] |
| Alemu et al. | 2019 | Primary school children | 5-14 years | Microscopic examination | Ethiopia | 351 | 17 | 10 | [91] |
| Mohamed et al. | 2019 | School Children | 6-15 years | Microscopic examination | Libya | 1110 | 20 | 10 | [92] |
| Saaed and Ongerth | 2019 | Children with diarrhea | 2-17 years | Microscopic examination | Libya | 505 | 133 | 10 | [93] |
| Jones et al. | 2019 | Preschool children | 0-72 month | Microscopic examination | Malawi | 193 | 24 | 8 | [94] |
| Butera et al. | 2019 | Children under 2 years | <2 years | Microscopic examination | Rwanda | 353 | 31 | 10 | [95] |
| Belkessa et al. | 2020 | Children | <15 years | Real time PCR assay | Algeria | 55 | 45 | 7 | [96] |
| Kurenzvi et al. | 2020 | Children with diarrhea | <5 years | Microscopic/ELISA/PCR | Botswana | 200 | 33 | 9 | [97] |
| Ndifor et al. | 2020 | Children 7m-7y | 7m-7 years | Microscopic examination | Chad | 200 | 21 | 9 | [98] |
| Abdisa et al. | 2020 | School Children | 7-13 years | Microscopic examination | Ethiopia | 384 | 18 | 10 | [99] |
| Mohamed et al. | 2020 | Symptomatic Children | 0.11<-<15 years | Microscopic examination | Eygpt | 617 | 61 | 10 | [100] |
| Chege et al. | 2020 | School-going children | 8-13 years | PCR | Kenya | 248 | 4 | 9 | [101] |
| Ferreira et al. | 2020 | Children under 5 years | <5 years | rapid immunochromatographic test | Mozambique | 831 | 199 | 10 | [102] |
| Bauhofer et al. | 2020 | Children hospitalized with diarrhea | 0-168 month | Immunoassays | Mozambique | 983 | 95 | 10 | [103] |
| Aiemjoy et al. | 2020 | young Children | 0-4 years | Real time PCR assay | Niger | 86 | 56 | 7 | [104] |
| Hajissa et al. | 2020 | School Children | 6-17 years | Microscopic examination | Sudan | 170 | 23 | 8 | [105] |
| Tembo et al. | 2020 | Asymptomatic School going Children | 3-16 years | Microscopic examination | Zambia | 329 | 33 | 10 | [106] |
| Belkessa et al. | 2021 | Children | <15 years | Microscopic examination | Algeria | 239 | 66 | 9 | [107] |
| Dange and Alelign | 2021 | School Children | 5-10 years and >10 (children of 1-8 grade level) | Microscopic examination | Ethiopia | 280 | 54 | 9 | [108] |
| Beavogui et al. | 2021 | Children | <15 years | Microscopic examination | Guinea | 392 | 20 | 9 | [109] |
| Mero et al. | 2021 | Chlidren under 5 years | <5 years | Real time PCR assay | Guinea-Bissau | 429 | 159 | 10 | [110] |
| Bauhofer et al. | 2021 | Hospitalized <5years children with diarrhea | <5 years | ELISA | Mozambique | 274 | 25 | 9 | [111] |
| Maudica et al. | 2021 | symptomatic and asymptomatic schoolchildren | 3-14 years | Real time PCR assay | Mozambique | 1093 | 459 | 10 | [112] |
| Messa et al. | 2021 | Chlidren under 5 years | <5 years | Real time PCR assay | Mozambique | 3754 | 1029 | 10 | [113] |
| Ahmed and Sheishaa | 2022 | School Children | 6-18 years | Microscopic examination | Eygpt | 726 | 62 | 10 | [114] |

NR: not report

QA: quality assessment

**References**

1. Asfaw ST, Giotom L: **Malnutrition and enteric parasitoses among under-five children in Aynalem Village, Tigray**. *Ethiopian Journal of Health Development* 2000, **14**(1):67-75.

2. Ibrahim M, Odoemena D, Ibrahim M: **Intestinal helminth ic infestations among primary school children in Sokoto**. *Sahel Medical Journal* 2000, **3**(2):65.

3. Okaka C, Awharitoma A, Okonji J: **Gastrointestinal parasites of school children in Benin city, Nigeria**. *Iranian Journal of Public Health* 2000, **29**(1-4):1-12.

4. Diouf S, Diallo A, Camara B, Diagne I, Signate H, Sarr M, Fall M: **Intestinal parasitoses in children in rural Senegal (Khombole)**. *Médecine d'Afrique Noire* 2000, **47**(5):229-232.

5. Gascon J, Vargas M, Schellenberg D, Urassa H, Casals C, Kahigwa E, Aponte J, Mshinda H, Vila J: **Diarrhea in children under 5 years of age from Ifakara, Tanzania: a case-control study**. *Journal of clinical microbiology* 2000, **38**(12):4459-4462.

6. Ukpai O, Ugwa C: **The prevalence of gastro-intestinal tract parasites in primary school children in Ikwuano Local Government Area of Abia State, Nigeria**. *Nigerian Journal of Parasitology* 2003, **24**(1):129-136.

7. Rice J, Skull SA, Pearce C, Mulholland N, Davie G, Carapetis J: **Screening for intestinal parasites in recently arrived children from East Africa**. *Journal of paediatrics and child health* 2003, **39**(6):456-459.

8. Legesse M, Erko B: **Prevalence of intestinal parasites among schoolchildren in a rural area close to the southeast of Lake Langano, Ethiopia**. *The Ethiopian Journal of Health Development* 2004, **18**(2).

9. Megahed A, Omar EA, Al-Sayed NA, Hassan MHA, El-Sahn AA: **Transmission dynamics of Giardia lamblia in a cohort of rural school children**. *Journal of High Institute of Public Health* 2005, **35**(2):233-254.

10. Itah AY, Opara K, Atting I, Udoidung N: **Prevalence of enteropathogens and their association with diarrhea among children of food vendors in Uyo, Nigeria**. *Mary Slessor Journal of Medicine* 2005, **5**(1):11-21.

11. Adamu H, Endeshaw T, Teka T, Kifle A, Petros B: **The prevalence of intestinal parasites in paediatric diarrhoeal and non-diarrhoeal patients in Addis Ababa hospitals, with special emphasis on opportunistic parasitic infections and with insight into the demographic and socio-economic factors**. *Ethiopian Journal of Health Development* 2006, **20**(1):39-46.

12. Aida A AM, Abdulla M E: **Factors associated with intestinal parasitic infection among school children in Sirt, Libya**. 2006.

13. El-Masry H, Ahmed Y, Hassan A, Zaky S, Abd-Allah E, El-Moselhy E, Abdel-Rahem M: **Prevalence, risk factors and impacts of schistosomal and intestinal parasitic infections among rural school children in Sohag Governorate**. *The Egyptian Journal of Hospital Medicine* 2007, **29**(1):616-630.

14. NA BM: **Intestinal parasites in school aged children and the first case report on amoebiasis in urinary bladder in Tripoli, Libya**. *Journal of the Egyptian Society of Parasitology* 2007, **37**(3):775-784.

15. Kasssem HH, Zaed HA, Sadaga GA: **Intestinal parasitic infection among children and neonatus admitted to Ibn-Sina Hospital, Sirt, Libya**. *Journal of the Egyptian Society of Parasitology* 2007, **37**(2):371-380.

16. El-Sherbini GT, Noor MA, Hegazi MM: **Parasitiosis in handicapped children in an Egyptian blind asylum**. *J Egypt Soc Parasitol* 2008, **38**(1):319-326.

17. Faiza A AES, Rasha A S, Nabila S T: **Predictors of the intestinal parasitic infection among pre-school children in rural lower, Egypt**. 2009.

18. Atia AH: **Prevalence of intestinal parasites among children and old patients in Alexandria Nahia**. *Journal of Techniques* 2009, **22**(2).

19. Worku N, Erko B, Torben W, Belay M, Kasssu A, Fetene T, Huruy K: **Malnutrition and intestinal parasitic infections in school children of Gondar, North West Ethiopia**. *Ethiopian medical journal* 2009, **47**(1):9-16.

20. Nyamngee A, Kalowole O, Durowade K, Kalowole C: **Prevalence of giardiasis among children in Guma Refugee camp in Guma LGA, Benue state, Nigeria**. *San Mateo Daily Journal* 2009, **6**(1):32-39.

21. Ogbolu D, Anorue M, Alli OT, Olaosun I, Olusoga-Ogbolu F: **Asymptomatic intestinal parasites in school children at Ota, Ogun State**. *African Journal of Biomedical Research* 2009, **12**(3):181-185.

22. Lalle M, Bruschi F, Castagna B, Campa M, Pozio E, Cacciò SM: **High genetic polymorphism among Giardia duodenalis isolates from Sahrawi children**. *Transactions of the Royal Society of Tropical Medicine and Hygiene* 2009, **103**(8):834-838.

23. Abdel aziz M A, Azam A A: **Intestinal protozoa and intestinal helminthic infections among schoolchildren in El dhayga, Central Sudan**. 2009.

24. Ouattara M, N'guéssan NA, Yapi A, N'goran EK: **Prevalence and spatial distribution of Entamoeba histolytica/dispar and Giardia lamblia among schoolchildren in Agboville area (Côte d'Ivoire)**. *PLoS neglected tropical diseases* 2010, **4**(1):e574.

25. Nyantekyi LA, Legesse M, Belay M, Tadesse K, Manaye K, Macias C, Erko B: **Intestinal parasitic infections among under-five children and maternal awareness about the infections in Shesha Kekele, Wondo Genet, Southern Ethiopia**. *Ethiopian Journal of Health Development* 2010, **24**(3).

26. Tigabu E, Petros B, Endeshaw T: **Prevalence of giardiasis and cryptosporidiosis among children in relation to water sources in selected village of Pawi Special District in Benishangul-Gumuz Region, northwestern Ethiopia**. *Ethiopian Journal of Health Development* 2010, **24**(3).

27. Abdel-Aziz MA, Afifi AA, Malik EM, Adam I: **Intestinal protozoa and intestinal helminthic infections among schoolchildren in Central Sudan**. *Asian Pacific Journal of Tropical Medicine* 2010, **3**(4):292-293.

28. Nitiema LW, Nordgren J, Ouermi D, Dianou D, Traore AS, Svensson L, Simpore J: **Burden of rotavirus and other enteropathogens among children with diarrhea in Burkina Faso**. *International Journal of Infectious Diseases* 2011, **15**(9):e646-e652.

29. Ayalew A, Debebe T, Worku A: **Prevalence and risk factors of intestinal parasites among Delgi school children, North Gondar, Ethiopia**. *J Parasitol Vector Biol* 2011, **3**(5):75-81.

30. Nkrumah B, Nguah SB: **Giardia lamblia: a major parasitic cause of childhood diarrhoea in patients attending a district hospital in Ghana**. *Parasites & vectors* 2011, **4**(1):1-7.

31. Langat B: **Spatial distribution of Giardia intestinalis in children up to 5 years old attending out-patient clinic at Provincial General hospital, Embu**. *Research Journal of Parasitology* 2011, **6**(4):136-143.

32. Inabo H, Ya’u B, Yakubu S: **Asymptomatic giardiasis and nutritional status of children in two local government areas in Kaduna State, Nigeria**. *Sierra Leone Journal of Biomedical Research* 2011, **3**(3):157-162.

33. Siwila J, Phiri IG, Enemark HL, Nchito M, Olsen A: **Seasonal prevalence and incidence of Cryptosporidium spp. and Giardia duodenalis and associated diarrhoea in children attending pre-school in Kafue, Zambia**. *Transactions of the Royal Society of Tropical Medicine and Hygiene* 2011, **105**(2):102-108.

34. Abdel-Hafeez EH, Ahmad AK, Ali BA, Moslam FA: **Opportunistic parasites among immunosuppressed children in Minia District, Egypt**. *The Korean journal of parasitology* 2012, **50**(1):57-62.

35. Mohammad K, Mohammad A, Abu El-Nour M, Saad MY, Timsah AG: **The prevalence and associated risk factors of intestinal parasitic infections among school children living in rural and urban communities in Damietta Governorate, Egypt**. *Academia Arena* 2012, **4**(5):90-97.

36. Krumkamp R, Acquah S, Adu-Sarkodie Y, Adelkofer J, Sarpong N, Jäger A, Tannich E, May J: **Gastrointestinal infections in Ghanaian children–disease agents and associated symptoms**. *International Journal of Infectious Diseases* 2012, **16**:e200.

37. Ferreira FS, Centeno-Lima S, Gomes J, Rosa F, Rosado V, Parreira R, Cravo L, Atouguia J, Távora Tavira L: **Molecular characterization of Giardia duodenalis in children from the Cufada Lagoon Natural Park, Guinea-Bissau**. *Parasitology research* 2012, **111**:2173-2177.

38. Opara KN, Udoidung NI, Opara DC, Okon OE, Edosomwan EU, Udoh AJ: **The impact of intestinal parasitic infections on the nutritional status of rural and urban school-aged children in Nigeria**. *International Journal of MCH and AIDS* 2012, **1**(1):73.

39. Ignatius R, Gahutu JB, Klotz C, Steininger C, Shyirambere C, Lyng M, Musemakweri A, Aebischer T, Martus P, Harms G: **High prevalence of Giardia duodenalis Assemblage B infection and association with underweight in Rwandan children**. *PLoS neglected tropical diseases* 2012, **6**(6):e1677.

40. M Mohamed M, I Ahmed A, T Salah E: **Frequency of intestinal parasitic infections among displaced children in Kassala Town**. *Khartoum medical journal* 2012, **2**(1).

41. Sadek GS, El-Settawy MA, Nasr SA: **Genotypic characterization of Giardia duodenalis in children in Menoufiya and Sharkiya governorates, Egypt**. *Life Sci J* 2013, **10**(1):3006-3015.

42. Unasho A: **An investigation of intestinal parasitic infections among the asymptomatic children in, Southern Ethiopia**. *International Journal* 2013, **2**(3):213.

43. Centeno-Lima S, Rosado-Marques V, Ferreira F, Rodrigues R, Indeque B, Camará I, de SOUSA B, Aguiar P, Nunes B, Ferrinho P: **Giardia Duodenalis e Desnutrição Crónica em Crianças Menores de Cinco Anos de uma Região Rural da Guiné-Bissau**. *Acta Médica Portuguesa* 2013, **26**(6):721-724.

44. Mbae CK, Nokes DJ, Mulinge E, Nyambura J, Waruru A, Kariuki S: **Intestinal parasitic infections in children presenting with diarrhoea in outpatient and inpatient settings in an informal settlement of Nairobi, Kenya**. *BMC Infect Dis* 2013, **13**:243.

45. Daw A, Al-Mubrook SA, Mhamed MC, Khan AH: **Prevalence of Giardiasis among Children from Wadi Al-Shati, Libya**. 2013.

46. Nxasana N, Baba K, Bhat V, Vasaikar S: **Prevalence of intestinal parasites in primary school children of Mthatha, Eastern Cape Province, South Africa**. *Annals of medical and health sciences Research* 2013, **3**(4):511-516.

47. Fusi-Ngwa C, Besong E, Pone JW, Mbida M: **A cross-sectional study of intestinal parasitic infections in children in Ghettoed, diverse and affluent communities in Dschang, west region, Cameroon**. *Open Access Library Journal* 2014, **1**(9):1-14.

48. Koffi M, N’Djeti M, Konan T, Djè Y: **Molecular characterization of intestinal protozoan parasites from children facing diarrheal disease and associated risk factors in Yamoussoukro, Côte d’Ivoire**. *African Journal of Environmental Science and Technology* 2014, **8**(3):178-184.

49. Helmy YA, Klotz C, Wilking H, Krücken J, Nöckler K, Samson-Himmelstjerna V, Zessin K-H, Aebischer T: **Epidemiology of Giardia duodenalis infection in ruminant livestock and children in the Ismailia province of Egypt: insights by genetic characterization**. *Parasites & Vectors* 2014, **7**(1):1-11.

50. El-Tantawy NL, Taman AI: **The epidemiology of Giardia intestinalis assemblages A and B among Egyptian children with diarrhea: A PCR-RFLP-based approach**. *Parasitologists United Journal* 2014, **7**(2):104.

51. Hegazy AM, Younis NT, Aminou HA, Badr AM: **Prevalence of intestinal parasites and its impact on nutritional status among preschool children living in Damanhur City, El-Behera Governorate, Egypt**. *Journal of the Egyptian Society of Parasitology* 2014, **44**(2):517-524.

52. Firdu T, Abunna F, Girma M: **Intestinal protozoal parasites in diarrheal children and associated risk factors at Yirgalem Hospital, Ethiopia: A case-control study**. *International scholarly research notices* 2014, **2014**.

53. Asemahagn MA: **Parasitic infection and associated factors among the primary school children in Motta town, western Amhara, Ethiopia**. *American Journal of Public Health Research* 2014, **2**(6):248-254.

54. Abossie A, Seid M: **Assessment of the prevalence of intestinal parasitosis and associated risk factors among primary school children in Chencha town, Southern Ethiopia**. *BMC public health* 2014, **14**(1):1-8.

55. Walana W, Tay SCK, Tetteh P, Ziem JB: **Prevalence of intestinal protozoan infestation among primary school children in urban and peri-urban communities in Kumasi, Ghana**. 2014.

56. Pavlinac PB, John-Stewart GC, Naulikha JM, Onchiri FM, Denno DM, Odundo EA, Singa BO, Richardson BA, Walson JL: **High-risk enteric pathogens associated with HIV-infection and HIV-exposure in Kenyan children with acute diarrhea**. *AIDS (London, England)* 2014, **28**(15):2287.

57. Messaad SA, Laboudi M, Moumni M, Sarhane B, Belghyti D, El Karrim K: **Children Intestinal parasites related to socio-economic factors in Salé Hospital, Morocco**. *International Journal of Innovation and Applied Studies* 2014, **8**(2):833.

58. El Fatni C, El Fatni H, Romero D, Olmo F, Rosales M: **Intestinal parasitism in Moroccan children: comparative quantitative study of the Faust’s and Ritchie’s coprologic methods**. *International Journal of Innovation and Applied Studies* 2014, **11**(1):53-64.

59. El Fatni C, Olmo F, El Fatni H, Romero D, Rosales MJ: **First genotyping of Giardia duodenalis and prevalence of enteroparasites in children from Tetouan (Morocco)**. *Parasite* 2014, **21**.

60. Fonseca AM, Fernandes N, Ferreira FS, Gomes J, Centeno-Lima S: **Intestinal parasites in children hospitalized at the Central Hospital in Maputo, Mozambique**. *The Journal of Infection in Developing Countries* 2014, **8**(06):786-789.

61. Ignatius R, Gahutu J, Klotz C, Musemakweri A, Aebischer T, Mockenhaupt F: **Detection of Giardia duodenalis assemblage A and B isolates by immunochromatography in stool samples from Rwandan children**. *Clinical Microbiology and Infection* 2014, **20**(10):O783-O785.

62. Danquah I, Gahutu JB, Ignatius R, Musemakweri A, Mockenhaupt FP: **Reduced prevalence of Giardia duodenalis in iron‐deficient Rwandan children**. *Tropical Medicine & International Health* 2014, **19**(5):563-567.

63. Lobo ML, Augusto J, Antunes F, Ceita J, Xiao L, Codices V, Matos O: **Cryptosporidium spp., Giardia duodenalis, Enterocytozoon bieneusi and other intestinal parasites in young children in Lobata province, Democratic Republic of São Tomé and Principe**. *PLoS One* 2014, **9**(5):e97708.

64. Gabbad AA, Elawad MA: **Prevalence of intestinal parasite infection in primary school children in Elengaz area, Khartoum, Sudan**. *Academic Research International* 2014, **5**(2):86.

65. Oliveira D, Ferreira FS, Atouguia J, Fortes F, Guerra A, Centeno-Lima S: **Infection by intestinal parasites, stunting and anemia in school-aged children from southern Angola**. *PloS one* 2015, **10**(9):e0137327.

66. Al Ghwass MM, El Dash HH, Amin SA, Hussin SS: **Intestinal parasitic infections and atopic diseases in children: a hospital based study**. *Journal of the Egyptian Society of Parasitology* 2015, **45**(2):413-419.

67. Mulatu G, Zeynudin A, Zemene E, Debalke S, Beyene G: **Intestinal parasitic infections among children under five years of age presenting with diarrhoeal diseases to two public health facilities in Hawassa, South Ethiopia**. *Infectious diseases of poverty* 2015, **4**:1-8.

68. Aleka Y, G/egziabher S, Tamir W, Birhane M, Alemu A: **Prevalence and associated risk factors of intestinal parasitic infection among under five children in University of Gondar Hospital, Gondar, Northwest Ethiopia**. *Biomedical Research and Therapy* 2015, **2**:1-7.

69. Yones DA, Galal LA, Abdallah AM, Zaghlol KS: **Effect of enteric parasitic infection on serum trace elements and nutritional status in upper Egyptian children**. *Tropical parasitology* 2015, **5**(1):29.

70. Ugochi UJ, Ifenyinwa M, Ijeoma E-N, Godson UM, Nwaku AI: **Prevalence of intestinal parasites among primary school children in three geopolitical zones of imo state, Nigeria**. *Science Journal of public health* 2015, **3**(5-1):25-28.

71. Saeed A, Abd H, Sandstrom G: **Microbial aetiology of acute diarrhoea in children under five years of age in Khartoum, Sudan**. *J Med Microbiol* 2015, **64**(Pt 4):432-437.

72. Ngosso BE, Nkwengulila G, Namkinga LA: **Identification of Pathogenic Intestinal Parasitic Protozoa Associated with Diarrhea among Under-fives Children in Dar Es Salaam, Tanzania**. In*: 2015*; 2015.

73. Tellevik MG, Moyo SJ, Blomberg B, Hjøllo T, Maselle SY, Langeland N, Hanevik K: **Prevalence of Cryptosporidium parvum/hominis, Entamoeba histolytica and Giardia lamblia among Young Children with and without Diarrhea in Dar es Salaam, Tanzania**. *PLoS Negl Trop Dis* 2015, **9**(10):e0004125.

74. Erismann S, Diagbouga S, Odermatt P, Knoblauch AM, Gerold J, Shrestha A, Grissoum T, Kaboré A, Schindler C, Utzinger J *et al*: **Prevalence of intestinal parasitic infections and associated risk factors among schoolchildren in the Plateau Central and Centre-Ouest regions of Burkina Faso**. *Parasites & Vectors* 2016, **9**(1):554.

75. Breurec S, Vanel N, Bata P, Chartier L, Farra A, Favennec L, Franck T, Giles-Vernick T, Gody JC, Luong Nguyen LB *et al*: **Etiology and Epidemiology of Diarrhea in Hospitalized Children from Low Income Country: A Matched Case-Control Study in Central African Republic**. *PLoS Negl Trop Dis* 2016, **10**(1):e0004283.

76. de Lucio A, Amor-Aramendía A, Bailo B, Saugar JM, Anegagrie M, Arroyo A, López-Quintana B, Zewdie D, Ayehubizu Z, Yizengaw E *et al*: **Prevalence and Genetic Diversity of Giardia duodenalis and Cryptosporidium spp. among School Children in a Rural Area of the Amhara Region, North-West Ethiopia**. *PLoS One* 2016, **11**(7):e0159992.

77. Madbouly NA, Farid A, El-Badry AA, El-Amir AM: **IMMUNE-MOLECULAR IDENTIFICATION OF GIARDIA INTESTINALIS IN DIARRHOEAL CHILDREN: COMPARISON OF THREE DIAGNOSTIC METHODS**. *J Egypt Soc Parasitol* 2016, **46**(2):253-260.

78. Monib MEM, Hassan A, Attia RAEH, Khalifa MM: **Prevalence of Intestinal Parasites among Children Attending Assiut University Children’s Hospital, Assiut, Egypt**. In*: 2016*; 2016.

79. Mbae C, Mulinge E, Guleid F, Wainaina J, Waruru A, Njiru ZK, Kariuki S: **Molecular Characterization of Giardia duodenalis in Children in Kenya**. *BMC Infect Dis* 2016, **16**:135.

80. Aiemjoy K, Gebresillasie S, Stoller NE, Shiferaw A, Tadesse Z, Chanyalew M, Aragie S, Callahan K, Keenan JD: **Epidemiology of Soil-Transmitted Helminth and Intestinal Protozoan Infections in Preschool-Aged Children in the Amhara Region of Ethiopia**. *Am J Trop Med Hyg* 2017, **96**(4):866-872.

81. El-Badry A, Mohammed F, Abdul Gawad E: **Predominance of Giardia intestinalis assemblage B in diarrhoeic children in Sharkia, Egypt**. *Parasitologists United Journal* 2017, **10**(1-2):39-43.

82. Forson AO, Arthur I, Olu-Taiwo M, Glover KK, Pappoe-Ashong PJ, Ayeh-Kumi PF: **Intestinal parasitic infections and risk factors: a cross-sectional survey of some school children in a suburb in Accra, Ghana**. *BMC research notes* 2017, **10**:1-5.

83. Esiet ULP, Edet I: **Comparative prevalence of intestinal parasites among children in public and private schools in Calabar South, Calabar, Cross River State, Nigeria**. *American Journal of Research Communication* 2017, **5**(1):80-97.

84. Siddig HS, Mohammed IA, Mohammed MN, Bashir AM: **Prevalence of intestinal parasites among selected group of primary school children in Alhag Yousif Area, Khartoum, Sudan**. *Int J Med Res Health Sci* 2017, **6**(8):125-131.

85. Abd Elhafiz M, Hajissa K, Mohamed Z, Aal AAA: **Prevalence of intestinal parasitic infection among children in al-kalakla, Khartoum, Sudan**. *World Applied Sciences Journal* 2017, **35**(2):219-222.

86. Feleke H, Medhin G, Abebe A, Beyene B, Kloos H, Asrat D: **Enteric pathogens and associated risk factors among under-five children with and without diarrhea in Wegera District, Northwestern Ethiopia**. *Pan African Medical Journal* 2018, **29**(1):1-10.

87. Naguib D, El-Gohary AH, Roellig D, Mohamed AA, Arafat N, Wang Y, Feng Y, Xiao L: **Molecular characterization of Cryptosporidium spp. and Giardia duodenalis in children in Egypt**. *Parasites & vectors* 2018, **11**(1):1-9.

88. Ibrahim HM, Salem AH, Arbi SA: **Prevalence of Entamoeba histolytica/Entamoeba dispar and Giardia lamblia infections among children in Sebha and Mourzak cities, Libya**. *Journal of Pure & Applied Sciences* 2018, **17**(1).

89. Huibers MH, Moons P, Maseko N, Gushu MB, Iwajomo OH, Heyderman RS, van Hensbroek MB, Brienen EA, van Lieshout L, Calis JC: **Multiplex real-time PCR detection of intestinal protozoa in HIV-infected children in Malawi, Enterocytozoon bieneusi is common and associated with gastrointestinal complaints and may delay BMI (nutritional status) recovery**. *The Pediatric infectious disease journal* 2018, **37**(9):910.

90. Mekonnen HS, Ekubagewargies DT: **Prevalence and factors associated with intestinal parasites among under-five children attending Woreta Health Center, Northwest Ethiopia**. *BMC infectious diseases* 2019, **19**(1):1-8.

91. Alemu G, Abossie A, Yohannes Z: **Current status of intestinal parasitic infections and associated factors among primary school children in Birbir town, Southern Ethiopia**. *BMC infectious diseases* 2019, **19**(1):1-8.

92. Mohamed MC: **Prevalence of Giardia lamblia among School Children in Brack Region, South Libya**. 2019.

93. Saaed FM, Ongerth JE: **Giardia and Cryptosporidium in children with diarrhea, Kufra, Libya, a North African migration route city**. *International journal of hygiene and environmental health* 2019, **222**(5):840-846.

94. Jones TP, Hart JD, Kalua K, Bailey RL: **A prevalence survey of enteral parasites in preschool children in the Mangochi District of Malawi**. *BMC infectious diseases* 2019, **19**:1-11.

95. Butera E, Mukabutera A, Nsereko E, Munyanshongore C, Rujeni N, Mwikarago IE, Moreland PJ, Manasse MN: **Prevalence and risk factors of intestinal parasites among children under two years of age in a rural area of Rutsiro district, Rwanda–a cross-sectional study**. *Pan African Medical Journal* 2019, **32**(1).

96. Belkessa S, Thomas-Lopez D, Houali K, Ghalmi F, Stensvold CR: **Molecular characterization of Giardia duodenalis in children and adults sampled in Algeria**. *Microorganisms* 2020, **9**(1):54.

97. Kurenzvi L, Sebunya TK, Coetzee T, Paganotti GM, Teye MV: **Prevalence of Cryptosporidium parvum, Giardia intestinalis and molecular characterization of group A rotavirus associated with diarrhea in children below five years old in Gaborone, Botswana**. *The Pan African Medical Journal* 2020, **37**.

98. Ndifor F, Alio HM, Adam IR, Lawane AI, Lhangadang F: **Giardia intestinalis and Entamoeba histolytica: Their Prevalence and Hematological Effects on Children between Seven Months to Seven Years at the Mother and Child Teaching Hospital in N’Djamena, Chad**.

99. Abdisa A, Dufera M, Nekemte E: **Study on Intestinal Parasitic Infections: Emphasis on Protozoan Parasites Entamoeba histolytica and Giardia lamblia and Associated Risk Factors Among School Children in Some Selected Primary Schools of Dembi Dolo Town, Western Ethiopia**. 2020.

100. Mohamed AM, Bayoumy AM, Abo-Hashim AH, Ibrahim AA, El-Badry AA: **Giardiasis in symptomatic children from Sharkia, Egypt: genetic assemblages and associated risk factors**. *Journal of Parasitic Diseases* 2020, **44**:719-724.

101. Chege N: **The prevalence of intestinal parasites and associated risk factors in school-going children from informal settlements in Nakuru town, Kenya**. *Malawi Medical Journal* 2020, **32**(2):80-86.

102. Ferreira FS, Pereira FdLM, Martins MdRO: **Intestinal parasitic infections in children under five in the Central Hospital of Nampula, Northern Mozambique**. *The Journal of Infection in Developing Countries* 2020, **14**(05):532-539.

103. Bauhofer AFL, Cossa-Moiane I, Marques S, Guimaraes EL, Munlela B, Anapakala E, Chilaule JJ, Cassocera M, Langa JS, Chissaque A: **Intestinal protozoan infections among children 0-168 months with diarrhea in Mozambique: June 2014-January 2018**. *PLoS neglected tropical diseases* 2020, **14**(4):e0008195.

104. Aiemjoy K, Arzika AM, Cook C, Lebas E, Pilotte N, Grant JR, Williams SA, Lietman TM, Keenan JD: **Molecular detection of intestinal helminths and protozoa among young children in Dosso Region, Niger**. *Gates Open Research* 2020, **4**(38):38.

105. Hajissa K, Abd Elhafiz M, Abd All T, Zakeia M, Eshag HA, Elnzer E, Nahied E, Jafer M, Sabo A, Mohamed Z: **Prevalence of Entamoeba histolytica and Giardia lamblia among schoolchildren in Um-Asher Area, Sudan**. 2020.

106. Tembo SJ, Mutengo MM, Sitali L, Changula K, Takada A, Mweene AS, Simulundu E, Chitanga S: **Prevalence and genotypic characterization of Giardia duodenalis isolates from asymptomatic school-going children in Lusaka, Zambia**. *Food and waterborne parasitology* 2020, **19**:e00072.

107. Belkessa S, Ait-Salem E, Laatamna A, Houali K, Sönksen UW, Hakem A, Bouchene Z, Ghalmi F, Stensvold CR: **Prevalence and clinical manifestations of Giardia intestinalis and other intestinal parasites in children and adults in Algeria**. *The American journal of tropical medicine and hygiene* 2021, **104**(3):910.

108. Dagne N, Alelign A: **Prevalence of Intestinal Protozoan Parasites and Associated Risk Factors among School Children in Merhabete District, Central Ethiopia**. *Journal of Parasitology Research* 2021, **2021**.

109. Beavogui AH, Cherif MS, Camara BS, Delamou A, Kolie D, Cissé A, Camara D, Sow A, Camara G, Yattara M: **Prevalence of parasitic infections in children of Boke, Guinea**. *The Journal of Parasitology* 2021, **107**(5):783-789.

110. Mero S, Timonen S, Lääveri T, Løfberg S, Kirveskari J, Ursing J, Rombo L, Kofoed P-E, Kantele A: **Prevalence of diarrhoeal pathogens among children under five years of age with and without diarrhoea in Guinea-Bissau**. *PLoS Neglected Tropical Diseases* 2021, **15**(9):e0009709.

111. Bauhofer AFL, Cossa-Moiane ILC, Marques SDA, Guimarães ELAM, Munlela BA, Anapakala EM, Chiláule JJ, Cassocera M, Langa JS, Chissaque A: **Intestinal protozoa in hospitalized under-five children with diarrhoea in Nampula–a cross-sectional analysis in a low-income setting in northern Mozambique**. *BMC Infectious Diseases* 2021, **21**(1):1-8.

112. Muadica AS, Köster PC, Dashti A, Bailo B, Hernández-de-Mingo M, Balasegaram S, Carmena D: **Molecular diversity of Giardia duodenalis, Cryptosporidium spp., and Blastocystis sp. in symptomatic and asymptomatic schoolchildren in Zambézia Province (Mozambique)**. *Pathogens* 2021, **10**(3):255.

113. Messa Jr A, Köster PC, Garrine M, Gilchrist C, Bartelt LA, Nhampossa T, Massora S, Kotloff K, Levine MM, Alonso PL: **Molecular diversity of Giardia duodenalis in children under 5 years from the Manhiça district, Southern Mozambique enrolled in a matched case-control study on the aetiology of diarrhoea**. *PLoS Neglected Tropical Diseases* 2021, **15**(1):e0008987.

114. Ahmed HM, Abu-Sheishaa GA: **Intestinal parasitic infection among school children in Dakahlia governorate, Egypt: a cross-sectional study**. *Egyptian Pediatric Association Gazette* 2022, **70**(1):1-8.
