# Supplementary material for: Prevalence of Giardia duodenalis among African children: A systematic review and meta-analysis
Source: Parasite Epidemiol Control. 2024 Jul 4;26:e00365. doi: 10.1016/j.parepi.2024.e00365 (PMC11277988; doi:10.1016/j.parepi.2024.e00365)
Supplement: Supplementary file 2 — Supplementary Figure 1. Results of sensitivity analysis based on individual study removal for estimating the prevalence of G. duodenalis in children. [file mmc2.docx]

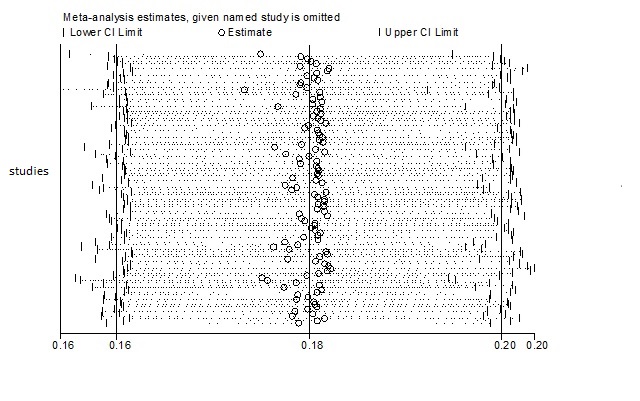


Supplementary Figure 1. Results of sensitivity analysis based on individual study removal for estimating the prevalence of *G. duodenalis* in children.
